# The impact of patient travel time on disparities in treatment for early stage lung cancer in California

**DOI:** 10.1371/journal.pone.0272076

**Published:** 2022-10-05

**Authors:** Chelsea A. Obrochta, Humberto Parada, James D. Murphy, Atsushi Nara, Dennis Trinidad, Maria Rosario (Happy) Araneta, Caroline A. Thompson

**Affiliations:** 1 San Diego State University, School of Public Health, San Diego, California, United States of America; 2 University of California San Diego, School of Medicine, La Jolla, California, United States of America; 3 University of California San Diego, Moores Cancer Center, La Jolla, California, United States of America; 4 Department of Geography, San Diego State University, San Diego, California, United States of America; 5 Department of Epidemiology, University of North Carolina Gillings School of Global Public Health, Chapel Hill, North Carolina, United States of America; Drexel University Dornsife School of Public Health, UNITED STATES

## Abstract

**Background:**

Travel time to treatment facilities may impede the receipt of guideline-concordant treatment (GCT) among patients diagnosed with early-stage non-small cell lung cancer (ES-NSCLC). We investigated the relative contribution of travel time in the receipt of GCT among ES-NSCLC patients.

**Methods:**

We included 22,821 ES-NSCLC patients diagnosed in California from 2006–2015. GCT was defined using the 2016 National Comprehensive Cancer Network guidelines, and delayed treatment was defined as treatment initiation >6 versus ≤6 weeks after diagnosis. Mean-centered driving and public transit times were calculated from patients’ residential block group centroid to the treatment facilities. We used logistic regression to estimate risk ratios and 95% confidence intervals (CIs) for the associations between patients’ travel time and receipt of GCT and timely treatment, overall and by race/ethnicity and neighborhood socioeconomic status (nSES).

**Results:**

Overall, a 15-minute increase in travel time was associated with a decreased risk of undertreatment and delayed treatment. Compared to Whites, among Blacks, a 15-minute increase in driving time was associated with a 24% (95%CI = 8%-42%) increased risk of undertreatment, and among Filipinos, a 15-minute increase in public transit time was associated with a 27% (95%CI = 13%-42%) increased risk of delayed treatment. Compared to the highest nSES, among the lowest nSES, 15-minute increases in driving and public transit times were associated with 33% (95%CI = 16%-52%) and 27% (95%CI = 16%-39%) increases in the risk of undertreatment and delayed treatment, respectively.

**Conclusion:**

The benefit of GCT observed with increased travel times may be a ‘Travel Time Paradox,’ and may vary across racial/ethnic and socioeconomic groups.

## Introduction

Favorable early-stage non-small cell lung cancer (NSCLC) prognosis is highly dependent on receipt of timely guideline-concordant treatment (GCT) [1]. Disparities in receipt of GCT have been observed among racial/ethnic minorities, those living in lower socioeconomic neighborhoods, and rural populations. An increased travel burden is associated with an increased diagnostic interval, more advanced disease at diagnosis, worse prognosis, and worse quality of life [2–27], as well as nonadherence to GCT [28] including undertreatment with surgery, radiation, chemotherapy, and adjuvant chemotherapy [4, 11, 19, 29–40]. However, the reported relationships between travel burden and cancer outcomes have been inconsistent. In previous studies, an increased travel burden was associated with a more rapid cancer diagnosis, lower overall mortality, and increased survival [40–43], while other studies show no association between travel burden and stage at diagnosis, treatment type, or long-term outcome [33, 44–47]. One study reported that women traveling farther distances to receive mastectomies were doing so after bypassing local options [20]; suggesting that an increased travel distance may be by choice, for some.

Receipt of cancer care may be influenced by a high travel burden as a result of residing long distances from treatment facilities or lack of private transportation. A higher travel burden has been documented for patients without a driver’s license or private vehicle [48] and for rural residents and non-Caucasians [44, 49–52]. On average, travel times are longer for public transportation compared to a private vehicle [49], however, there is some evidence that treatment facilities are favorably located closer to neighborhoods with the lowest household access to a private vehicle [50].

The objective of this study was to investigate the relative contribution of patients’ travel times to their treatment facilities on racial/ethnic and socioeconomic disparities in receipt of GCT among patients diagnosed with early-stage NSCLC in California. As higher travel burden has been observed in minority and lower socioeconomic groups, we hypothesized that the effect of travel time to treatment facilities on GCT differs by race/ethnicity and neighborhood socioeconomic status (nSES).

## Methods

### Data source

The California Cancer Registry (CCR) is a statewide population-based cancer surveillance program [53]. By law, all occurrences of cancer among Californians are required to be reported to the CCR, ensuring the population is representative of all of California [53]. Cancer details, demographics, and social and clinical details were collected by the CCR. County 2013 rural-urban continuum codes were ascertained from the United States (U.S.) Department of Agriculture. To determine the location of a patient’s cancer treatment facility, a list of complete addresses was compiled using Google and geocoded in ArcGIS PRO 2.4.

This study was reviewed and approved by Institutional Review Boards (IRBs) at San Diego State University, the University of California San Diego, and the California Department of Public Health Committee for the Protection of Human Subjects.

### Study population

We included 23,571 patients diagnosed with first primary, stages I-II, NSCLC, as defined by the American Joint Committee of Cancer 7th edition, between 2006 and 2015, and alive at the time of diagnosis. Of these, we excluded patients due to the following reasons: missing lymph node (N) staging (n = 122) or missing date of diagnosis (n = 127), which were required to determine receipt of GCT; missing race (n = 43) or those who were classified as multiracial (n = 288) or other Hispanics (n = 9) due to race being required to assess differences by race, no validated methods to analyze multiracial categories, and a small sample size of other Hispanics; transsexual or transgender (n = 4) individuals due to small sample sizes; missing residential census block group (n = 20), missing treatment facility (n = 68), or requiring a ferry for transit/driving time incalculable (n = 3), which were required to determine travel times; driving distance >250 miles (n = 66), which were outliers for travel times. After applying these exclusions, the final study population comprised 22,821 patients.

### Assessment of GCT

The primary outcome was receipt of GCT according to the 2016 National Comprehensive Cancer Network (NCCN) guidelines defined as the administration of proper initial and adjuvant surgery, chemotherapy, or radiation treatment(s) according to cancer site and stage. If a patient did not receive surgery, they were assumed inoperable and assessed for GCT according to lymph node staging (N0 or N1). Alternatively, undertreatment was less than minimum site- and stage- specific recommended treatment.

**Fig 1:**
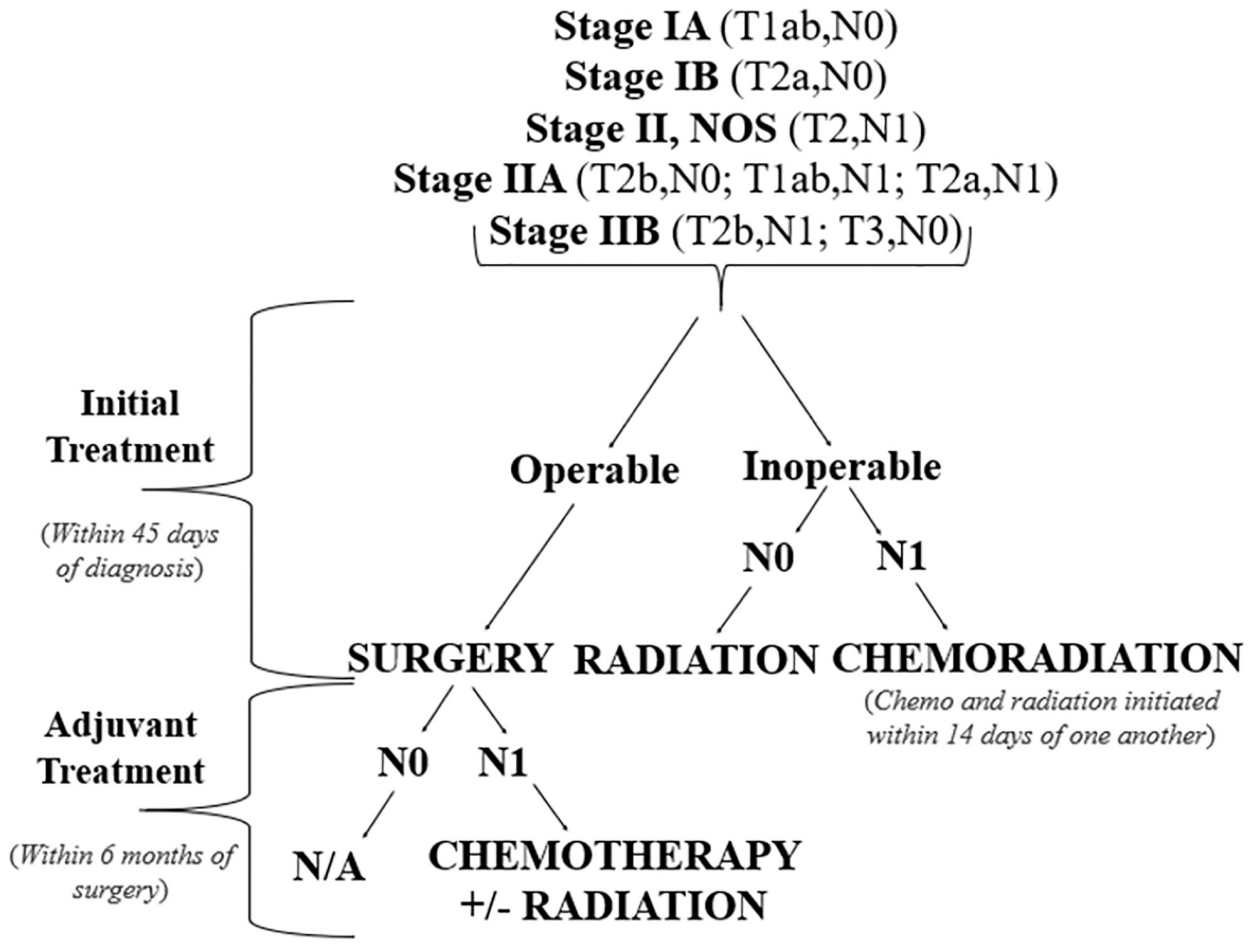
NSCLC GCT based on NCCN guidelines. NSCLC recommended treatment according to the 2016 NCCN guidelines. For stage 1A-IIB NSCLC, an operable patients’ initial treatment should be surgery within 45 days of diagnosis. If the patient is node 1 (N1), adjuvant treatment of chemotherapy +/- radiation should be administered within 6 months of surgery. For inoperable patients, initial treatment differs by lymph node involvement. If the patient is node 0 (N0), initial treatment should be radiation within 45 days of diagnosis. If the patient is node 1 (N1), initial treatment should be chemoradiation within 45 days of diagnosis. If a patients’ chemotherapy and radiation start date are within 2 weeks of one another, this will be considered chemoradiation.

The secondary outcome was receipt of timely (versus delayed) GCT. The Research ANd Development Corporation recommends treatment initiation within 6 weeks of diagnosis [54] (i.e., the initiation of surgery, radiation, or chemotherapy within 45 days of diagnosis), and The Commission of Cancer Quality of Care Measures recommends adjuvant treatment of chemotherapy administration within 6 months of surgery, when required [55] (i.e., the initiation of chemotherapy +/- radiation within 6 months of initial surgery for N1 patients); Fig 1.

To determine receipt of GCT and timely treatment, full dates for diagnosis, surgery, radiation, and chemotherapy are required. If only month and year were available, the middle of the month day was imputed.

### Assessment of travel time

Mean-centered travel time [56, 57] to treatment facilities including driving and public transit travel times (minutes) to a patient’s chosen treatment facility from their residence was calculated from the centroid of their census block group [58]. ArcGIS Online’s *Connect Origins to Destinations* Analysis was used to compute driving travel time based on historical and live traffic data [59]. Public transportation was calculable for 11,607 patients living in census blocks with transit service available (nearest transit stop within 0.75 miles). The Google Maps Application Programming Interface with the *gmapsdistance* function in R was used to compute public transit travel time; *gmapsdistance* requires a future travel time and was specified as an arrival date and time of Monday, October 9^th^, 2020 at 5pm; 5pm was chosen to account for less traffic during the COVID-19 pandemic. Driving time was also calculated using *gmapsdistance* with the same specifications to compare the two methods of calculating driving travel time.

### Effect modifiers

Patient race/ethnicity and nSES were investigated as potential effect modifiers of the association between travel time and receipt of GCT. Race/ethnicity was classified as non-Hispanic White (NHW), non-Hispanic Black (NHB), Hispanic (including those who identify as white or Black), Native Hawaiian and Pacific Islander (NHPI), Chinese, Japanese, Filipino, Korean, Vietnamese, Asian Indian, Other Asian, or American Indian. Race/ethnicity data in the CCR is based on hospital records that use self-report, assumptions of hospital personnel, or extrapolation from birthplace, race/ethnicity of parent, maiden name, or surname [60]. nSES in the CCR is determined from the American Community Survey using a composite residential neighborhood-level index that combines census measures of education, income, occupation, and cost of living at the census block group level and categorized into quintiles [61].

### Covariates

Covariates included stage at diagnosis [IA (T1ab,N0), IB (T2a,N0), II, NOS (T2,N1), IIA (T2b,N0; T1ab,N1; T2a,N1), IIB (T2b,N1; T3,N0)], year of diagnosis, sex, age, insurance type (not insured, private insurance, Medicaid, Medicare, military, other/not otherwise specified), marital status (single/never married, partnered (married/unmarried or domestic partner), unpartnered (separated/divorced, widowed)), whether or not the reporting facility with the earliest date of admission had an ACOS-approved cancer program, and rural-urban continuum codes. Rural-urban continuum codes (1–9) distinguishes metropolitan counties by the population of their metro area, and nonmetropolitan counties by degree of urbanization and adjacency to a metro area are assigned to each county [62]. To resolve unavailability of payer (n = 298), marital status (n = 564), and cancer program (n = 46) information, we used multiple imputation, a valid statistical procedure for recovering missing data to create complete datasets that can then be analyzed through standard procedures [63].

### Statistical analysis

**Fig 2:**
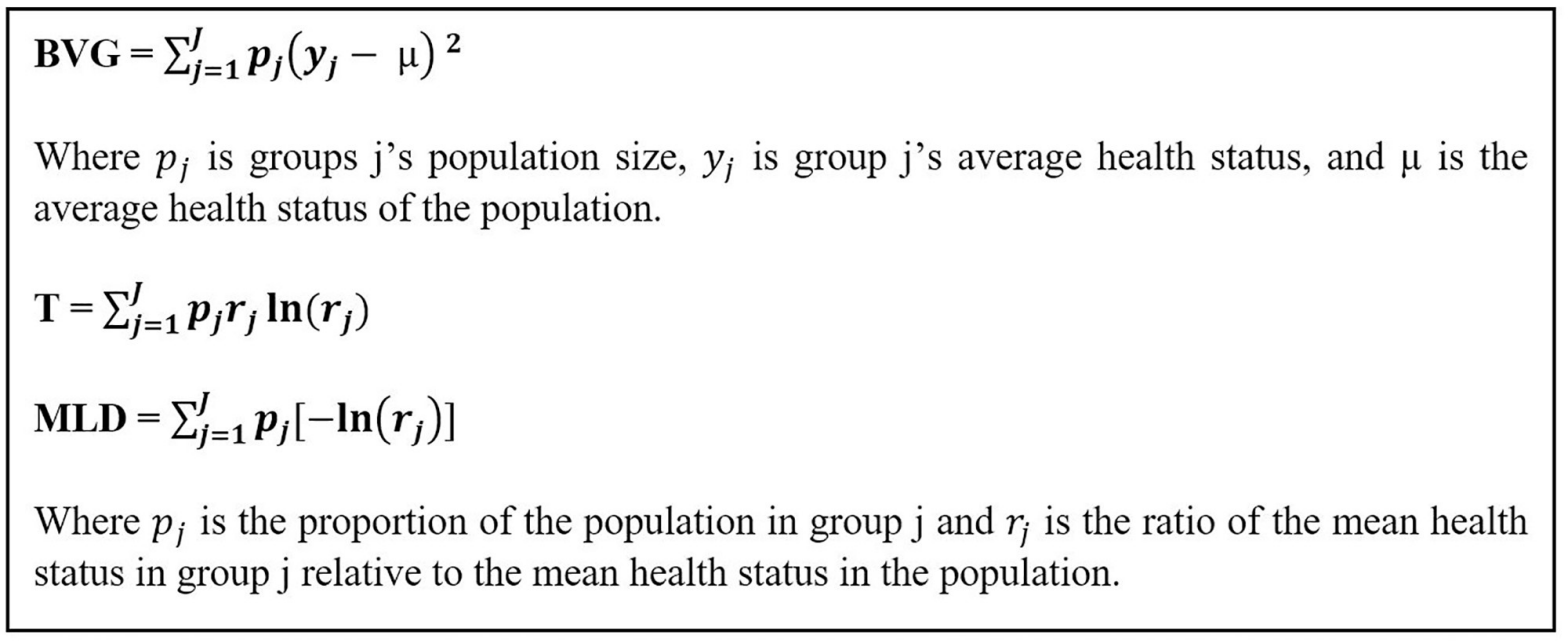
Absolute and relative disparities measure formulas. Average disproportionality in receipt of GCT and timely treatment is measured using three disproportionality functions: Between-Groups Variance (BGV), The Theil Index (T), and Mean Log Deviation (MLD). BGV is a useful metric of absolute disparity for unordered groups. T and MLD are entropy-based measures that quantify the relative disparity. T and MLD are complementary measures.

Exposure, clinical and sociodemographic information were stratified by race/ethnicity. We quantified average disproportionality in receipt of GCT and timely treatment across categories of race/ethnicity, nSES, and driving and public transit travel times (<15, 15–30, 30–60, and ≥60 minutes) using three disproportionality functions: Between-Groups Variance (BGV), The Theil Index (T), and Mean Log Deviation (MLD). BGV is a useful metric of absolute disparity for unordered groups, such as race/ethnicity, because it weights by population size and is sensitive to larger deviations from the population average. T and MLD are entropy-based measures that quantify the relative disparity, meaning the disproportionate receipt of GCT and timely GCT across effect modifiers and exposures. T and MLD are complementary measures because T can be influenced by groups with high ratios of GCT and timely GCT in a group relative to the average GCT and timely GCT in the population, and MLD can be influenced by groups with larger population shares [64]; formulas provided in Fig 2.

We used multivariable generalized logistic regression models (PROC GENMOD) with a Poisson distribution and log link function to explore all combinations of the following associations: outcomes (undertreatment and delayed GCT), exposures (mean-centered driving and public transit travel time), and effect modifiers (race/ethnicity and nSES), to estimate the impact of travel time to treatment facilities on both racial/ethnic and socioeconomic disparities in undertreatment and delayed GCT. The intraclass correlation coefficient (ICC) of treatment hospital was assessed to determine if treatment hospital needed to be included as a random effect. Driving and public transit travel times were mean-centered and scaled to represent a 15-minute increase from the population average. Patient racial/ethnic groups with less than 100 persons (NHPI, Asian Indian, and American Indian) were excluded from models due to small sample sizes. In addition to disaggregating Asian groups with sufficient sample sizes, an aggregated Asian American, Native Hawaiian, and Pacific Islander (AANHPI) models was run separately including NHPIs and Asian Indians. Overall, we had 28 covariate-adjusted models. Models 1, 8, 15, and 22 regressed the outcomes (undertreatment and delayed GCT) on the effect modifiers (race/ethnicity and nSES). Models 2, 5, 9, 12, 16, 23, and 26 regressed the outcomes (undertreatment and delayed GCT) on the exposures (driving and public transit time). Models 3, 6, 10, 13, 17, 20, 24, and 27 combined the above models. Models 4, 7, 11, 14, 18, 21, 25, and 28 extended the previous models by adding an interaction term between the effect modifiers (race/ethnicity and nSES), and the exposures (driving and public transit time). The interaction models were the primary models of interest. nSES was not adjusted for when considering race/ethnicity as an effect modifier, but race/ethnicity was adjusted for when considering nSES as an effect modifier. Risk Ratios (RR) and 95% Confidence Intervals (CI) for the effect measure modifier analyses are presented in Table 4, while the betas and 95% CIs for all 28 models are available in (S1 Table, effect modifier: race/ethnicity; S2 Table, effect modifier: nSES). A sensitivity analysis considering driving time calculated using the *gmapsdistance* function were compared to the above results. All analyses were performed using SAS version 9.4 (SAS Institute Inc., Cary, NC).

## Results

Among the 22,821 early-stage NSCLC patients, 18,471 (80.94%) received GCT and, of these, 10,632 (57.56%) received timely GCT. Exposure, clinical and sociodemographic characteristics, stratified by race/ethnicity, are displayed in Table 1. Cells counts <5 are suppressed.

**Table 1:** Exposure variables and clinical and sociodemographic characteristics, stratified by patient race/ethnicity.

	All	Patient Race/Ethnicity											
		non-Hispanic White (n = 16450)	non-Hispanic Black (n = 1463)	Hispanic (n = 2263)	NHPI (n = 66)	Chinese (n = 771)	Japanese (n = 180)	Filipino (n = 632)	Korean (n = 195)	Vietnamese (n = 360)	Asian Indian (n = 84)	Other Asian (n = 325)	American Indian (n = 32)
**Exposure Variables**	n (%) or *Mean (SD)												
Driving Travel Times*	26.0 (26.5)	26.8 (28.0)	23.2 (20.7)	25.1 (24.6)	25.1 (25.4)	23.0 (18.9)	20.9 (14.5)	23.9 (22.7)	27.1 (23.3)	21.6 (15.0)	29.4 (29.2)	22.1 (19.5)	26.9 (25.3)
< 15 minutes	8703 (38.1)	6296 (38.3)	570 (39.0)	871 (38.5)	26 (39.4)	285 (37.0)	67 (37.2)	240 (38.0)	65 (33.3)	118 (32.8)	28 (33.3)	127 (39.1)	10 (31.3)
15–30 minutes	8345 (36.6)	5843 (35.5)	578 (39.5)	838 (37.0)	27 (40.9)	301 (39.0)	85 (47.2)	250 (39.6)	64 (32.8)	186 (51.7)	28 (33.3)	131 (40.3)	14 (43.8)
30–60 minutes	4033 (17.7)	2896 (17.6)	252 (17.2)	409 (18.1)	8 (12.1)	158 (20.5)	23 (12.8)	110 (17.4)	47 (24.1)	46 (12.8)	21 (25.0)	57 (17.5)	6 (18.8)
≥ 60 minutes	1740 (7.6)	1415 (8.6)	63 (4.3)	145 (6.4)	5 (7.6)	27 (3.5)	5 (2.8)	32 (5.1)	19 (9.7)	10 (2.8)	7 (8.3)	10 (3.1)	--
Public Transit Travel Times*	68.6 (66.2)	71.3 (70.6)	60.5 (45.7)	67.2 (68.3)	64.6 (50.8)	54.7 (49.9)	61.2 (34.7)	65.4 (49.9)	76.5 (89.9)	62.4 (39.1)	96.4 (90.4)	61.8 (34.3)	64.6 (32.5)
< 15 minutes	476 (2.1)	302 (1.8)	48 (3.3)	47 (2.1)	--	37 (4.8)	--	18 (2.9)	5 (2.6)	6 (1.7)	--	6 (1.9)	--
15–30 minutes	1891 (8.3)	1241 (7.5)	183 (12.5)	191 (8.4)	10 (15.2)	114 (14.8)	15 (8.3)	42 (6.7)	24 (12.3)	39 (10.8)	--	29 (8.9)	--
30–60 minutes	4186 (18.3)	2692 (16.4)	417 (28.5)	473 (20.9)	14 (21.2)	197 (25.6)	42 (23.3)	133 (21.0)	43 (22.1)	95 (26.4)	12 (14.3)	67 (20.6)	--
≥ 60 minutes	5054 (22.2)	3411 (20.7)	452 (30.9)	508 (22.5)	15 (22.7)	179 (23.2)	43 (23.9)	169 (26.7)	65 (33.3)	97 (26.9)	22 (26.2)	83 (25.5)	10 (31.3)
Unavailable	11214 (49.1)	8804 (53.5)	363 (24.8)	1044 (46.1)	27 (40.9)	244 (31.7)	76 (42.2)	270 (42.7)	58 (29.7)	123 (34.2)	47 (56.0)	140 (43.1)	18 (56.3)
**Clinical and Sociodemographic Characteristics**	n (%) or *Mean (SD)												
***Stage***													
IA	10522 (46.1)	7720 (46.9)	619 (42.3)	1008 (44.5)	30 (45.5)	333 (43.2)	74 (41.1)	277 (43.8)	78 (40.0)	172 (47.8)	31 (36.9)	164 (50.5)	16 (50.0)
IB	7259 (31.8)	5182 (31.5)	452 (30.9)	738 (32.6)	20 (30.3)	263 (34.1)	65 (36.1)	218 (34.5)	70 (35.9)	113 (31.4)	38 (45.2)	89 (27.4)	11 (34.4)
II	35 (0.2)	21 (0.1)	--	--	--	--	--	--	--	--	--	--	--
IIA	2458 (10.8)	1718 (10.4)	171 (11.7)	251 (11.1)	7 (10.6)	101 (13.1)	19 (10.6)	76 (12.0)	28 (14.4)	35 (9.7)	8 (9.5)	41 (12.6)	--
IIB	2547 (11.2)	1809 (11.0)	218 (14.9)	262 (11.6)	9 (13.6)	71 (9.2)	22 (12.2)	58 (9.2)	19 (9.7)	40 (11.1)	7 (8.3)	30 (9.2)	--
Year of diagnosis*	2010.7 (2.9)	2010.6 (2.9)	2010.8 (2.8)	2010.9 (2.8)	2011.4 (2.9)	2010.9 (2.9)	2010.1 (2.8)	2011.0 (2.8)	2011.0 (2.7)	2011.0 (2.9)	2011.2 (2.7)	2011.7 (2.7)	2011.7 (2.6)
2006–2010	10760 (47.2)	8003 (48.7)	50 (3.4)	990 (43.8)	27 (40.9)	333 (43.2)	98 (54.4)	266 (42.1)	87 (44.6)	145 (40.3)	34 (40.5)	100 (30.8)	13 (40.6)
2011–2015	12061 (52.9)	8447 (51.4)	451 (30.8)	1273 (56.3)	39 (59.1)	438 (56.8)	82 (45.6)	366 (57.9)	108 (55.4)	215 (59.7)	50 (59.5)	225 (69.2)	19 (59.4)
***Sex***													
Male	10383 (45.5)	7390 (44.9)	645 (44.1)	978 (43.2)	32 (48.5)	415 (53.8)	64 (35.6)	316 (50.0)	113 (58.0)	214 (59.4)	46 (54.8)	154 (47.4)	16 (50.0)
Female	12438 (54.5)	9060 (55.1)	818 (55.9)	1285 (56.8)	34 (51.5)	356 (46.2)	116 (64.4)	316 (50.0)	82 (42.1)	146 (40.6)	38 (45.2)	171 (52.6)	16 (50.0)
Age groups*	70.4 (10.7)	71.0 (10.3)	67.1 (10.6)	69.1 (12.3)	67.1 (10.6)	70.1 (11.0)	74.2 (10.0)	70.1 (10.4)	69.2 (9.5)	67.6 (11.2)	67.1 (12.6)	68.9 (12.3)	68.2 (13.8)
18 through 45	394 (1.7)	200 (1.2)	329 (22.5)	95 (4.2)	--	16 (2.1)	--	10 (1.6)	5 (2.6)	11 (3.1)	9 (10.7)	14 (4.3)	--
46 through 60	3453 (15.1)	2284 (13.9)	(0.0)	381 (16.8)	18 (27.3)	126 (16.3)	18 (10.0)	100 (15.8)	29 (14.9)	78 (21.7)	12 (14.3)	60 (18.5)	10 (31.3)
61 through 75	11169 (48.9)	8100 (49.2)	664 (45.4)	1042 (46.1)	32 (48.5)	352 (45.7)	67 (37.2)	323 (51.1)	108 (55.4)	178 (49.4)	43 (51.2)	148 (45.5)	10 (31.3)
76 +	7805 (34.2)	5866 (35.7)	799 (54.6)	745 (32.9)	15 (22.7)	277 (35.9)	94 (52.2)	199 (31.5)	53 (27.2)	93 (25.8)	20 (23.8)	103 (31.7)	11 (34.4)
**Payer**													
Not insured	155 (0.7)	85 (0.5)	14 (1.0)	31 (1.4)	--	--	--	6 (1.0)	5 (2.6)	--	--	7 (2.2)	--
Private	8493 (37.2)	6064 (36.9)	568 (38.8)	818 (36.2)	21 (31.8)	339 (44.0)	77 (42.8)	270 (42.7)	54 (27.7)	101 (28.1)	31 (36.9)	137 (42.2)	13 (40.6)
Medicaid	1097 (4.8)	543 (3.3)	165 (11.3)	188 (8.3)	7 (10.6)	51 (6.6)	--	58 (9.2)	13 (6.7)	34 (9.4)	9 (10.7)	28 (8.6)	--
Medicare	11946 (52.4)	8962 (54.5)	632 (43.2)	1115 (49.3)	34 (51.5)	324 (42.0)	97 (53.9)	275 (43.5)	116 (59.5)	198 (55.0)	36 (42.9)	143 (44.0)	14 (43.8)
Military	104 (0.5)	81 (0.5)	8 (0.6)	8 (0.4)	--	--	--	--	--	--	--	--	--
Other or NOS	728 (3.2)	492 (3.0)	55 (3.7)	76 (3.3)	--	45 (5.8)	5 (2.8)	13 (2.1)	5 (2.6)	22 (6.1)	--	8 (2.5)	--
*Missing*	298 (1.3)	223 (1.4)	21 (1.4)	27 (1.2)	--	--	--	7 (1.1)	--	--	--	--	--
***Marital Status at diagnosis***													
Single	564 (2.5)	2097 (12.8)	451 (30.38)	321 (14.2)	9 (13.6)	48 (6.2)	16 (8.9)	37 (5.9)	12 (6.2)	35 (9.7)	6 (7.1)	31 (9.5)	5 (15.6)
Partnered	12091 (53.0)	8567 (52.1)	504 (34.5)	1181 (52.2)	39 (59.1)	574 (74.5)	112 (62.2)	429 (67.9)	142 (72.8)	261 (72.5)	67 (79.8)	201 (61.9)	14 (43.8)
Unpartnered	3068 (13.4)	5391 (32.8)	458 (31.31)	698 (30.8)	16 (24.2)	134 (17.4)	51 (28.3)	154 (24.4)	35 (18.0)	55 (15.3)	11 (13.1)	84 (25.9)	11 (34.4)
*Missing*	7098 (31.1)	395 (2.4)	50 (3.42)	63 (2.8)	--	15 (2.0)	--	12 (1.9)	6 (3.1)	9 (2.5)	6 (7.1)	9 (2.8)	--
***Cancer Program***													
Approved	13803 (60.5)	10179 (61.9)	751 (51.3)	1313 (58.0)	34 (51.5)	403 (52.3)	108 (60.0)	351 (55.5)	126 (64.6)	264 (73.3)	59 (70.2)	197 (60.6)	18 (56.3)
Not approved	8972 (39.3)	6240 (37.9)	709 (48.5)	948 (41.9)	31 (47.0)	366 (47.5)	70 (38.9)	278 (44.0)	68 (34.9)	95 (26.4)	25 (29.8)	128 (39.4)	14 (43.8)
*Missing*	46 (0.2)	31 (0.2)	--	--	--	--	--	--	--	--	--	--	--
***Neighborhood Social Economic Status***													
lowest	3243 (14.2)	1889 (11.5)	435 (29.7)	600 (26.5)	13 (19.7)	103 (13.4)	11 (6.1)	79 (12.5)	25 (12.8)	41 (11.4)	--	41 (12.6)	--
lower-middle	4494 (19.7)	3041 (18.5)	389 (26.6)	558 (24.7)	17 (25.8)	89 (11.5)	34 (18.9)	122 (19.3)	39 (20.0)	109 (30.3)	10 (11.9)	73 (22.5)	13 (40.6)
middle	4927 (21.6)	3568 (21.7)	309 (21.1)	508 (22.5)	14 (21.2)	130 (16.9)	41 (22.8)	161 (25.5)	35 (18.0)	84 (23.3)	14 (16.7)	57 (17.5)	6 (18.8)
upper-middle	5025 (22.0)	3845 (23.4)	214 (14.6)	363 (16.0)	12 (18.2)	186 (24.1)	46 (25.6)	162 (25.6)	37 (19.0)	67 (18.6)	20 (23.8)	66 (20.3)	7 (21.9)
highest	5132 (22.5)	4107 (25.0)	116 (7.9)	234 (10.3)	10 (15.2)	263 (34.1)	48 (26.7)	108 (17.1)	59 (30.3)	59 (16.4)	38 (45.2)	88 (27.1)	--
Rural-Urban Continuum Code*	1.4 (0.9)	1.5 (1.0)	1.1 (0.4)	1.3 (0.7)	1.3 (0.7)	1.0 (0.2)	1.2 (0.5)	1.2 (0.4)	1.1 (0.3)	1.0 (0.2)	1.3 (0.5)	1.1 (0.4)	1.9 (1.4)
1: Metro (1 million or more)	17007 (74.5)	11657 (70.9)	1283 (87.7)	1688 (74.6)	49 (74.2)	743 (96.4)	150 (83.3)	540 (85.4)	185 (94.9)	349 (96.9)	66 (78.6)	279 (85.9)	18 (56.3)
2: Metro (250,000 to 1 million)	4133 (18.1)	3271 (19.9)	163 (11.1)	466 (20.6)	13 (19.7)	25 (3.2)	29 (16.1)	81 (12.8)	9 (4.6)	10 (2.8)	15 (17.9)	44 (13.5)	7 (21.9)
3: Metro (fewer than 250,000 population	910 (4.0)	789 (4.8)	15 (1.0)	80 (3.5)	--	--	--	10 (1.6)	--	--	--	--	--
4: Nonmetro (20,000 or more, adjacent to a metro area)	426 (1.9)	406 (2.5)	--	18 (0.8)	--	--	--	--	--	--	--	--	--
5: Nonmetro (20,000 or more, not adjacent to a metro area)	105 (0.5)	101 (0.6)	--	--	--	--	--	--	--	--	--	--	--
6: Nonmetro (2,500 to 19,999, adjacent to a metro area)	166 (0.7)	155 (0.9)	--	8 (0.4)	--	--	--	--	--	--	--	--	--
7: Nonmetro (2,500 to 19,999, not adjacent to a metro area)	41 (0.2)	40 (0.2)	--	--	--	--	--	--	--	--	--	--	--
8: Nonmetro (less than 2,500)	33 (0.1%)	31 (0.2%)	--	--	--	--	--	--	--	--	--	--	--

— Cell counts < 5 suppressed.

### Clinical and sociodemographic characteristics

Stage at diagnosis varied by race/ethnicity with NHBs having the highest proportion of Stage IIB diagnosis (14.9%). Females accounted for 54.5% of patients overall, but 64.4% of Japanese and 40.6% Vietnamese patients. The mean age at diagnosis was 70.4 years overall and ranged from 67.1 years for NHPI to 74.2 years for Japanese patients. Less than 1% of patients were uninsured, and half were married or in a domestic partnership. Most patients were treated at hospitals with an ACOS-approved cancer program (60.5%) with lower rates among NHBs (51.3%), NHPIs (51.5%), and Chinese (52.3%). nSES differed by race/ethnicity; overall, 14.2% of patients lived in the lowest nSES, but NHBs (29.7%), Hispanics (26.5%), and NHPIs (19.7%) proportions were much higher, and most patients lived in metro areas.

### Travel time

The mean (μ) driving time was 26 (standard deviation(σ) = 26.5) minutes with NHWs (μ = 26.8), Koreans (μ = 27.1), Asian Indians (μ = 29.4), and American Indians (μ = 26.9) having longer driving times than the average. Half (49.1%) of the population had no public transportation available with unavailability more frequent among NHWs (53.5%), Asian Indians (56.0%), and American Indians (56.3%). Among patients with available public transportation, the mean public transit time was 68.6 (σ = 66.2) minutes with NHWs (μ = 71.3), Koreans (μ = 76.5), and Asian Indians (μ = 96.4) having longer than the average public transit times. Driving and public transit times, stratified by nSES, are provided in (S3 Table). Patients with the highest nSES have the shortest travel times.

### Absolute and relative disparity measures

The proportions of receipt of GCT ranged from 76.35% among NHBs to 84.70% among Chinese and the proportions of receipt of timely treatment ranged from 49.80% among Filipinos to 72.06% among Other Asians. Patient’s living in the highest nSES had the highest proportion of GCT (84.53%) and timely treatment (66.25%), followed by upper-middle, middle, lower-middle, and lowest SES (GCT = 75.33%; timely GCT = 50.43%) nSES (Table 2). Patients with a ≥60 minutes driving time had the highest percent GCT (86.90%) and timely treatment (64.95%), followed by 30–60, 15–30, and <15 minutes (GCT = 77.36%; timely treatment = 56.29%). Patients with a ≥60 minutes public transit time had the highest proportion of GCT (82.33%) and timely GCT (58.65%) (Table 3). BVG, Theil, and MLD values range from 0 to ∞ (higher inequality) and should be used to compare the level of inequality across outcomes and groups. We observed more absolute disparity in rate of timely GCT, compared to GCT, between race/ethnicity (GCT = 3.65; timely GCT = 8.65) and nSES (GCT = 10.10; timely GCT = 28.35), with higher absolute disparity in nSES compared to race/ethnicity. There was more absolute disparity in GCT (driving = 10.73; public transit = 8.60) compared to timely GCT (driving = 5.65; public transit = 2.18), between travel times. There was very little relative disparity in rate of GCT and timely GCT.

**Table 2:** Absolute and relative disparities in rate of GCT and Timely GCT between patient race/ethnicity groups and neighborhood socioeconomic status (nSES).

**GCT and Patient Race/Ethnicity (n = 22,821)**					
**Patient Race/Ethnicity**	**GCT (%)**	**Proportion of Population**	**BVG**	**Theil**	**MLD**
NHW (n = 16450)	81.75	0.7208	0.4729	0.0072	-0.0072
NHB (n = 1463)	76.35	0.0641	1.3505	-0.0035	0.0037
Hispanic (n = 2263)	77.60	0.0992	1.1066	-0.0040	0.0042
NHPI (n = 66)	78.79	0.0029	0.0134	-0.0001	0.0001
Chinese (n = 771)	84.70	0.0338	0.4779	0.0016	-0.0015
Japanese (n = 180)	82.78	0.0079	0.0267	0.0002	-0.0002
Filipino (n = 632)	80.70	0.0277	0.0016	-0.0001	0.0001
Korean (n = 195)	78.46	0.0085	0.0523	-0.0003	0.0003
Vietnamese (n = 360)	78.61	0.0158	0.0858	-0.0004	0.0005
Asian Indian (n = 84)	80.95	0.0037	0.0000	0.0000	0.0000
Other Asian (n = 325)	78.77	0.0142	0.0669	-0.0004	0.0004
American Indian (n = 32)	81.25	0.0014	0.0001	0.0000	0.0000
**All Groups**	**80.94**		**3.6547**	**0.0003**	**0.0003**
**Timely GCT and Patient Race/Ethnicity (n = 18,471)**					
**Patient Race/Ethnicity**	**Timely GCT (%)**	**Proportion of Population**	**BVG**	**Theil**	**MLD**
NHW (n = 13448)	58.43	0.7281	0.5511	0.0111	-0.0109
NHB (n = 1117)	50.04	0.0605	3.4213	-0.0074	0.0085
Hispanic (n = 1756)	54.78	0.0951	0.7350	-0.0045	0.0047
NHPI (n = 52)	65.38	0.0028	0.1712	0.0004	-0.0004
Chinese (n = 653)	60.34	0.0354	0.2736	0.0018	-0.0017
Japanese (n = 149)	57.72	0.0081	0.0002	0.0000	0.0000
Filipino (n = 510)	49.80	0.0276	1.6620	-0.0035	0.0040
Korean (n = 153)	61.44	0.0083	0.1250	0.0006	-0.0005
Vietnamese (n = 283)	56.89	0.0153	0.0069	-0.0002	0.0002
Asian Indian (n = 68)	72.06	0.0037	0.7779	0.0010	-0.0008
Other Asian (n = 256)	65.63	0.0139	0.9052	0.0021	-0.0018
American Indian (n = 26)	53.85	0.0014	0.0193	-0.0001	0.0001
**All Groups**	**57.56**		**8.6486**	**0.0014**	**0.0013**
**GCT and Neighborhood Socioeconomic Status (n = 22,821)**					
**nSES**	**GCT (%)**	**Proportion of Population**	**BVG**	**Theil**	**MLD**
Highest (n = 5132)	84.53	0.2249	2.8985	0.0102	-0.0098
Upper-Middle (n = 5025)	83.24	0.2202	1.1649	0.0063	-0.0062
Middle (n = 4927)	81.10	0.2159	0.0055	0.0004	-0.0004
Lower-Middle (n = 4494)	78.13	0.1969	1.5547	-0.0067	0.0070
Lowest (n = 3243)	75.33	0.1421	4.4722	-0.0095	0.0102
**All Groups**	**80.94**		**10.0958**	**0.0008**	**0.0008**
**Timely GCT and Neighborhood Socioeconomic Status (n = 18,471)**					
**nSES**	**Timely GCT (%)**	**Proportion of Population**	**BVG**	**Theil**	**MLD**
Highest (n = 4338)	66.25	0.2349	17.7387	0.0380	-0.0330
Upper-Middle (n = 4183)	57.95	0.2265	0.0345	0.0015	-0.0015
Middle (n = 3996)	55.56	0.2163	0.8652	-0.0074	0.0076
Lower-Middle (n = 3511)	53.60	0.1901	2.9811	-0.0126	0.0136
Lowest (n = 2443)	50.43	0.1323	6.7257	-0.0153	0.0175
**All Groups**	**57.56**		**28.3452**	**0.0042**	**0.0041**

**Table 3:** Absolute and relative disparities in rate of GCT and Timely GCT between driving travel time and public transit travel time.

**GCT and Driving Travel Time (n = 22,821)**					
**Driving Travel Time**	**GCT (%)**	**Proportion of Population**	**BVG**	**Theil**	**MLD**
< 15 minutes (n = 8703)	77.36	0.3814	4.8882	-0.0165	0.0173
15–30 minutes (n = 8345)	81.41	0.3657	0.0808	0.0021	-0.0021
30–60 minutes (n = 4033)	85.10	0.1767	3.0579	0.0093	-0.0089
≥ 60 minutes (n = 1740)	86.90	0.0762	2.7067	0.0058	-0.0054
**All Groups**	**80.94**		**10.7336**	**0.0008**	**0.0009**
**Timely GCT and Driving Travel Time (n = 18,471)**					
**Driving Travel Time**	**Timely GCT (%)**	**Proportion of Population**	**BVG**	**Theil**	**MLD**
< 15 minutes (n = 6733)	56.29	0.3645	0.5879	-0.0081	0.0081
15–30 minutes (n = 6794)	56.59	0.3678	0.3461	-0.0060	0.0063
30–60 minutes (n = 3432)	58.71	0.1858	0.2457	0.0038	-0.0037
≥ 60 minutes (n = 1512)	64.95	0.0819	4.4727	0.0111	-0.0099
**All Groups**	**57.56**		**5.6524**	**0.0008**	**0.0008**
**GCT and Public Transit Travel Time (n = 11,607)**					
**Public Transit Travel Time**	**GCT (%)**	**Proportion of Population**	**BVG**	**Theil**	**MLD**
< 15 minutes (n = 476)	77.73	0.0410	0.4225	-0.0009	0.0009
15–30 minutes (n = 1891)	76.15	0.1629	3.7376	-0.0067	0.0070
30–60 minutes (n = 4186)	77.78	0.3606	3.6008	-0.0077	0.0079
≥ 60 minutes (n = 5054)	82.33	0.4354	0.8412	0.0158	-0.0152
**All Groups**	**79.50**		**8.6021**	**0.0004**	**0.0006**
**Timely GCT and Public Transit Travel Time (n = 9,227)**					
**Public Transit Travel Time**	**Timely GCT (5)**	**Proportion of Population**	**BVG**	**Theil**	**MLD**
< 15 minutes (n = 370)	52.97	0.0401	0.8448	-0.0029	0.0031
15–30 minutes (n = 1440)	55.63	0.1561	0.5815	-0.0044	0.0046
30–60 minutes (n = 3256)	56.76	0.3529	0.2259	-0.0032	0.0032
≥ 60 minutes (n = 4161)	58.64	0.4510	0.5260	0.0108	-0.0106
**All Groups**	**57.28**		**2.1782**	**0.0003**	**0.0003**

To explain how driving and public transit time impacted the risk of undertreatment and delayed GCT, multivariable mean-centered models are described below. Treatment hospital had an intraclass correlation coefficient of < 5% and therefore was not included as a random effect.

#### Outcomes and effect modifiers

Compared to NHWs, NHBs (beta(β) = 0.21, 95%CI = 0.11–0.30), Hispanics (β = 0.20, 95%CI = 0.12–0.28), and Vietnamese (β = 0.34, 95%CI = 0.15–0.54) had higher risks for undertreatment, and NHBs (β = 0.15, 95%CI = 0.09–0.22), Hispanics (β = 0.08, 95%CI = 0.03–0.14), and Filipinos (β = 0.18, 95%CI = 0.10–0.27) had higher risk for delayed GCT (S1 Table). Compared to patients in the highest nSES, patients in the middle (β = 0.13, 95%CI = 0.05–0.21), lower-middle (β = 0.23, 95%CI = 0.15–0.31), and lowest (β = 0.30, 95%CI = 0.22–0.39) nSES had higher risk for undertreatment, and those in the upper-middle (β = 0.19, 95%CI = 0.14–0.25), middle (β = 0.24, 95%CI = 0.18–0.29), lower-middle (β = 0.27, 95%CI = 0.22–0.33), and lowest (β = 0.32, 95%CI = 0.26–0.38) nSES had higher risk for delayed GCT (S2 Table).

#### Outcomes and exposures

When considering all patients, a 15-minute increase (from the mean) in driving time was associated with a 5.48% (β = -0.06, 95%CI = -0.08,-0.04) and 3.10% (β = -0.03, 95%CI = -0.04,-0.02) decreased relative risk for undertreatment and delayed treatment, respectively, and a 15-minute increase in public transit times was associated with a 1.78% (β = -0.02, 95%CI = -0.03,-0.01) and 0.7% (β = -0.01, 95%CI = -0.01,0.00) decreased relative risk for undertreatment and delayed GCT, respectively (S2 Table). However, increased travel times did not translate to improved care for all racial/ethnic or socioeconomic groups as evidenced by our joint exposure models.

#### Outcomes, effect modifiers, exposures, and interactions

Considering a joint exposure that incorporates both travel time and race/ethnicity, a 15-minute increase in driving time for NHBs and Koreans increased their risk of undertreatment by 24% (95%CI = 8%-42%) and 37% (95%CI = 2%-82%), respectively, compared to NHWs. A 15-minutes increase in public transit time for NHBs, Hispanics, Vietnamese, and Other Asians increased their risk of undertreatment by 29% (95%CI = 14%-46%), 32% (95%CI = 16%-49%), 49% (95%CI = 15%-93%), and 39% (95%CI = 7%-82%) respectively, compared to NHWs. A 15-minute increase in driving time for NHBs and Filipinos increased their risk of delayed GCT by 17% (95%CI = 7%-28%) and 27% (95%CI = 15%-41%), respectively, compared to NHWs. A 15-minutes increase in public transit time for NHBs, Hispanics, and Filipinos increased their risk for of delayed GCT by 18% (95%CI = 9%-28%), 12% (95%CI = 4%-21%), and 27% (95%CI = 13%-42%), respectively, compared to NHWs (Table 4).

**Table 4:** Risk Ratios (RR) and 95 Confidence Intervals (CI) for race/ethnicity and neighborhood socioeconomic status (nSES) representing that effect as modified by a 15-minute increase in travel time.

	Outcome: Undertreatment				Outcome: Delayed GCT			
	Exposure: Driving Time		Exposure: Public Transit Time		Exposure: Driving Time		Exposure: Public Transit Time	
	Model 4 Summary^a^	Model 18 Summary^a^	Model 7 Summary^b^	Model 25 Summary^b^	Model 11 Summary^a^	Model 21 Summary^a^	Model 14 Summary^b^	Model 28 Summary^b^
Effect Modifier	RR (95 CI)				RR (95 CI)			
***Race/Ethnicity***								
**NHW**	REFERENCE		REFERENCE		REFERENCE		REFERENCE	
**NHB**	**1.24 (1.08,1.42)**		**1.29 (1.14,1.46)**		**1.17 (1.07,1.28)**		**1.18 (1.09,1.28)**	
**Hispanic**	1.11 (0.97,1.27)		**1.32 (1.16,1.49)**		1.06 (0.99,1.14)		**1.12 (1.04,1.21)**	
AANHPI*	1.04 (0.89,1.21)		1.10 (0.98,1.24)		1.00 (0.93,1.08)		1.07 (0.98,1.16)	
**Chinese**	0.93 (0.69,1.26)		0.85 (0.64, 1.14)		0.88 (0.75,1.03)		1.01 (0.85,1.21)	
**Japanese**	0.84 (0.52,1.34)		1.15 (0.80, 1.65)		1.14 (0.85,1.54)		1.18 (0.91,1.53)	
**Filipino**	1.09 (0.84,1.42)		1.06 (0.84, 1.33)		**1.27 (1.15,1.41)**		**1.27 (1.13,1.42)**	
**Korean**	**1.37 (1.02,1.82)**		1.04 (0.73, 1.48)		0.86 (0.62,1.19)		0.94 (0.73,1.23)	
**Vietnamese**	0.93 (0.55,1.57)		**1.49 (1.15, 1.93)**		0.96 (0.74,1.24)		1.02 (0.81,1.28)	
**Other Asian**	1.30 (0.97,1.75)		**1.39 (1.07, 1.82)**		0.87 (0.70,1.10)		0.87 (0.64,1.18)	
***nSES***								
**Highest**		REFERENCE		REFERENCE		REFERENCE		REFERENCE
**Upper-Middle**		1.06 (0.93,1.21)		1.05 (0.92,1.20)		**1.26 (1.16,1.36)**		**1.13 (1.04,1.23)**
**Middle**		1.12 (0.99,1.28)		**1.15 (1.01,1.30)**		**1.29 (1.20,1.40)**		**1.21 (1.11,1.32)**
**Lower-Middle**		**1.27 (1.12,1.44)**		**1.31 (1.16,1.49)**		**1.38 (1.28,1.49)**		**1.21 (1.11,1.32)**
**Lowest**		**1.33 (1.16,1.52)**		**1.39 (1.22,1.59)**		**1.44 (1.33,1.56)**		**1.27 (1.16,1.39)**
***P trend***		**< 0.0001**		**<0.0001**		**<0.0001**		**<0.0001**

*Separate model with aggregate AANHPI which include NHPI and Asian Indians.

^a^ Risk Ratio (Exponentiated Estimate) for Race/Ethnicity represents Race/Ethnicity effect (reference: non-Hispanic white with 15-minute increase in travel time) as modified by a 15-minute increase in travel time (with product term to capture effect modification by travel time, adjusted for age, year of diagnosis, stage at diagnosis, sex, insurance, marital status, cancer approved program, and rural-urban continuum code.

^b^ Risk Ratio (Exponentiated Estimates) for nSES represent nSES effects (reference: Highest nSES with 15-minute increase in travel time) as modified by a 15-minute increase in travel time (with product term to capture effect modification by travel time), adjusted for age, year of diagnosis, stage at diagnosis, sex, race/ethnicity, insurance, marital status, cancer approved program, and rural-urban continuum code

Considering a joint exposure that incorporates both travel time and nSES, a 15-minute increase in driving time for patients in the lower-middle and lowest nSES increased their risk of undertreatment by 27% (95%CI = 12%-44%) and 33% (95%CI = 16%-52%) compared to patients in the highest nSES (P-for-trend<0.01), respectively. A 15-minute increase in public transit time for patients in the lower-middle and lowest nSES increased their risk of undertreatment by 31% (95%CI = 16%-49%) and 39% (95%CI = 22%-59%), respectively, compared to patients in the highest nSES (P-for-trend<0.01). A 15-minute increase in driving time for patients in the upper-middle, middle, lower-middle, and lowest nSES increased their risk of delayed GCT by 26% (95%CI = 16%-36%) to 44% (95%CI = 33%-56%) compared to patients in the highest nSES (P-for-trend<0.01). A 15-minute increase in public transit time for patients in the upper-middle, middle, lower-middle, and lowest nSES increased their risk of delayed GCT by 13% (95%CI = 4%-23%) to 27% (95%CI = 16%-39%) compared to patients in the highest nSES (P-for-trend<0.01) (Table 4).

Sensitivity analyses considering driving time calculated using *gmapsdistance* were compared to the above results using ArcGIS Online’s *Connect Origins to Destinations* Analysis. Estimates differed slightly, but groups at significantly increased risk for undertreatment and delayed GCT were consistent (S4 Table).

## Discussion

Racial/ethnic and socioeconomic disparities in receipt of GCT and timely treatment exist among early-stage NSCLC patients in California. NHBs experienced the lowest rate of GCT and Filipinos and NHBs experienced the lowest rates of timely treatment, and patients living in the highest nSES experienced the highest rate of timely GCT with a linear decreasing trend with decreasing nSES. On average, a 15-minute increase in travel time was associated with a decreased risk for undertreatment and delayed treatment. This protective effect observed from increased travel times was unexpected and may be a ‘Travel Time Paradox,” but this paradox was not uniform across all groups.

NHBs and Hispanics were at higher relative risk as compared to Whites for undertreatment and delayed treatment. NHBs and Hispanics had shorter travel times and the highest proportions of patients in lower nSES. Interestingly, when considering the interaction between travel time and race/ethnicity, a 15-minute increase in driving time for Hispanics attenuated the risk of undertreatment and delayed treatment, compared to NHWs. This could be explained by healthcare facilities near Hispanic neighborhoods being poorer. Opposing, a 15-minute increase in public transit time for Hispanics increased the magnitude of risk of undertreatment and delayed treatment, compared to NHWs. It is unclear why this “Travel Time Paradox” would not hold in Hispanics for public transit, but it may be that patients requiring public transit are less likely to travel farther for better care when travel times are already three times longer than driving. Further, a 15-minute increase in driving and public transit time for NHBs increased risk of undertreatment and delayed treatment, compared to NHWs. This supports a racial/ethnic disparity that is not overcome by a farther, more qualified, healthcare facility.

In aggregate, AANHPIs were not at increased relative risk for undertreatment or delayed treatment, however, by disaggregated Asian groups important heterogeneity was illuminated. Compared to NHWs, Koreans and Vietnamese were at higher risk for undertreatment and Filipinos were at higher risk for delayed treatment. Filipinos and Vietnamese had shorter travel times and relatively average nSES. For Vietnamese, however, a 15-minute increase in driving time for Vietnamese appears to protect against undertreatment compared to NHWs and reveals the benefit for Vietnamese to travel farther for better cancer care. On the other hand, a 15-minute increase in public transit time for Vietnamese increases the risk of undertreatment, compared to NHWs. A 15-minute increase in driving and public transit time for Filipinos increases the risk of undertreatment and exaggerates the risk of delayed treatment, compared to NHWs. Lastly, Other Asians are at higher risk for undertreatment and lower risk for delayed treatment compared to NHWs, but a 15-minute increase in travel time significantly increases risk for undertreatment and delayed treatment, compared to NHWs.

We observed a linear relationship between increased travel time and risk of undertreatment and treatment delay by decreasing quintile of nSES. For patients in the lowest nSES, a 15-minute increase in travel time resulted in 33–39% and 27–44% increased risks of undertreatment and delayed treatment, respectively. This may be explained by lower socioeconomic patients not having as good of choices, even if traveling farther. Interestingly, a 15-minute increase in driving time for non-highest nSES patients increases the risk of delayed treatment and a 15-minute increase in public transit time for the non-highest nSES patients attenuates the risk of delayed treatment, compared to the highest nSES patients. This may be due to patients in lower nSES wanting to drive farther for better care, but it simply taking longer to find the time.

In previous U.S. studies [65–67], increased travel distance within urban areas decreased receipt of timely treatment, while within rural areas, the inverse relationship was found. These studies considered distance as opposed to time, which may have influenced results as driving the same distance in an urban setting likely takes longer than in a rural setting. Our public transit time results generally represent urban areas in which this ‘Travel Time Paradox’ holds, although attenuated compared to driving time, and contradictory to the above studies’ findings. Most other U.S. studies considered assessed travel distance as opposed to travel time, and found that increased travel distance decreased likelihood of treatment [28, 35–37].

This “Travel Time Paradox” has not been previously reported in U.S. patients. In one Australian study, early-stage NSCLC patients living farther away were less likely to have surgery and more likely to attend a general hospital rather than a specialist hospital. But, for patients that were treated in specialist hospitals, the relationship with distance was inverse showing a protective effect with longer distance [21]. Although our study is not directly comparable due to differences in healthcare systems, our study supports the hypothesis that patients may choose, if resources allow, to travel farther for better cancer care, and the closest hospital may not have the resources to provide proper treatment. Further, two recent U.S. studies showed that early-stage NSCLC who were treated at an academic facility compared to a community facility had significantly higher median overall survival, and Black patients were more likely to undergo surgery at academic facilities [68, 69]. Our study controlled for ACOS-approved cancer program to try and account for quality of care and the importance of facility type, but also found no random effect by treatment facility.

We considered a patient’s chosen treatment facility as opposed to the nearest facility, as often examined [14–23, 28, 32, 34, 36, 43, 49, 50, 65–67]. Considering the nearest treatment facility may make sense in countries with universal healthcare or clearly defined catchment regions, but this topic is much more complex in the U.S. where patients’ healthcare utilization is driven by insurance, choice, and convenience [70]. Thus, our observed ‘Travel Time Paradox’ may be driven by a patient’s choice to travel further for improved cancer care.

The findings from this study should be interpreted in light of the limitations. The CCR does not provide patient refusal or comorbidities preventing treatment which could result in outcome misclassification. Further, a patient’s ability to get appropriate care may be attributable to more than just proximity to care. One consideration is that wealthier patients may choose to travel farther for their cancer care than a poorer patient. We tried to unpack this by assessing nSES as an effect modifier, but due to limited sample sizes, we were unable to stratify our results by both race/ethnicity and nSES. A strength of this study includes the presentation of disaggregated Asian groups; aggregating Asians into one group masks heterogeneity between groups. Additionally, we consider a patient’s chosen treatment facility, as opposed to nearest treatment facility, and so our exposure is representative of the treatment facility a patient chose to attend.

These findings help elucidate the cancer-related health disparities within California’s highly diverse population. Undertreatment and delayed treatment for early-stage NSCLC disproportionately affect minorities and those living in lower socioeconomic status neighborhoods. The protective effect observed from increased travel times may be a ‘Travel Time Paradox’. This paradox effect may be partially explained by patients choosing to travel farther for better care or having to travel farther to receive treatment. However, a patient’s ability to travel farther for care could be prohibited for many reasons such as lack of time or personal transportation thus additional healthcare facilities may not be the solution. While cancer treatment transportation options may be beneficial to patients who lack a private vehicle [71, 72], accessible high-quality healthcare facilities that offer surgery, radiation, and chemotherapy are required.

## Supporting information

S1 Table Effect of travel time to treatment facilities on racial/ethnic disparities in undertreatment and delayed GCT. (DOCX)

S2 Table Effect of travel time to treatment facilities on socioeconomic disparities in undertreatment and delayed GCT. (DOCX)

S3 Table Driving and public transit times, stratified by neighborhood socioeconomic status. (DOCX)

S4 Table Sensitivity analysis: Risk Ratios (RR) and 95% Confidence Intervals (CI) for race/ethnicity and neighborhood socioeconomic status (nSES) representing that effect as modified by a 15-minute increase in driving time calculate using the gmapsdistance function. (DOCX)
