# Supplementary material for: The impact of patient travel time on disparities in treatment for early stage lung cancer in California
Source: PLoS One. 2022 Oct 5;17(10):e0272076. doi: 10.1371/journal.pone.0272076 (PMC9534452; doi:10.1371/journal.pone.0272076)
Supplement: S1 Table — (DOCX) [file pone.0272076.s001.docx]

**S1 Table.** **Effect of travel time to treatment facilities on racial/ethnic disparities in undertreatment and delayed GCT.** Betas and 95% CIs for Model 1 through Model 14 were adjusted for age, year of diagnosis, stage at diagnosis, sex, insurance, marital status, cancer approved program, and rural-urban continuum code. Model 1 regressed undertreatment on race/ethnicity. Model 2 regressed undertreatment on driving time. Model 3 combined Model 1 and Model 2. Model 4 extended Model 3 by adding an interaction term between race/ethnicity and driving time. Model 5 regressed undertreatment on public transit time. Model 6 combined Model 1 and Model 5. Model 7 extended Model 6 by adding an interaction term between race/ethnicity and public transit time. Model 8 regressed delayed GCT on race/ethnicity. Model 9 regressed delayed GCT on driving time. Model 10 combined Model 8 and Model 9. Model 11 extended Model 10 by adding an interaction term between race/ethnicity and driving time. Model 12 regressed delayed GCT on public transit time. Model 13 combined Model 8 and Model 12. Model 14 extended Model 13 by adding an interaction term between race/ethnicity and public transit time. Estimates for race represent race effects as modified by a 15-minute increase in travel time (with product term to capture effect modification by travel time).

| **Variable** | **Model 1^a^** | **Model 2^a^** | **Model 3^a^** | **Model 4^a^** | **Model 4 Summary^b^** | **Model 5^a^** | **Model 6^a^** | **Model 7^a^** | **Model 7 Summary^b^** |  |
| --- | --- | --- | --- | --- | --- | --- | --- | --- | --- | --- |
| **Outcome: Undertreatment Driving Travel Time n = 22,639; Public Transit Travel Time n = 11,517** | | | | | | | | | |  |
| ***Race*** |  |  |  |  |  |  |  |  |  |  |
| **NHW** | REFERENCE |  | REFERENCE | REFERENCE | REFERENCE |  | REFERENCE | REFERENCE | REFERENCE |  |
| **NHB** | **0.21 (0.11, 0.30)** |  | **0.20 (0.11, 0.30)** | **0.21 (0.11, 0.30)** | **0.21 (0.08, 0.35)** |  | **0.21 (0.10, 0.32)** | **0.23 (0.12, 0.34)** | **0.25 (0.13, 0.38)** |  |
| **Hispanic** | **0.20 (0.12, 0.28)** |  | **0.20 (0.12, 0.28)** | **0.18 (0.09, 0.26)** | 0.10 (-0.03, 0.24) |  | **0.29 (0.19, 0.40)** | **0.29 (0.18, 0.39)** | **0.28 (0.15, 0.40)** |  |
| **AANHPI*** | **0.10 (0.02, 0.18)** |  | 0.09 (0.01, 0.17) | 0.08 (0.00, 0.17) | 0.05 (-0.09, 0.20) |  | **0.13 (0.03, 0.23)** | **0.12 (0.01, 0.22)** | 0.10 (-0.02, 0.22) |  |
| **Chinese** | -0.12 (-0.28, 0.04) |  | -0.13 (-0.29, 0.03) | -0.11 (-0.29, 0.06) | -0.07 (-0.37, 0.23) |  | -0.09 (-0.27, 0.09) | -0.13 (-0.36, 0.10) | -0.16 (-0.45, 0.13) |  |
| **Japanese** | -0.15 (-0.44, 0.13) |  | -0.16 (-0.45, 0.12) | -0.17 (-0.47, 0.14) | -0.18 (-0.65, 0.29) |  | 0.09 (-0.23, 0.41) | 0.11 (-0.22, 0.43) | 0.14 (-0.23, 0.50) |  |
| **Filipino** | 0.13 (-0.02, 0.28) |  | 0.13 (-0.02, 0.28) | 0.12 (-0.04, 0.28) | 0.09 (-0.17, 0.35) |  | 0.12 (-0.08, 0.31) | 0.10 (-0.11, 0.30) | 0.06 (-0.17, 0.29) |  |
| **Korean** | **0.26 (0.01, 0.50)** |  | **0.27 (0.02, 0.51)** | **0.27 (0.02, 0.51)** | **0.31 (0.02, 0.60)** |  | 0.09 (-0.23, 0.42) | 0.08 (-0.25, 0.41) | 0.04 (-0.32, 0.39) |  |
| **Vietnamese** | **0.34 (0.15, 0.54)** |  | **0.33 (0.14, 0.53)** | 0.20 (-0.06, 0.47) | -0.07 (-0.59, 0.45) |  | **0.47 (0.26, 0.69)** | **0.44 (0.21, 0.66)** | **0.40 (0.14, 0.66)** |  |
| **Other Asian** | **0.21 (0.01, 0.41)** |  | 0.20 (0.00, 0.40) | **0.22 (0.01, 0.43)** | 0.26 (-0.03, 0.56) |  | 0.23 (-0.02, 0.48) | **0.27 (0.02, 0.51)** | **0.33 (0.07, 0.60)** |  |
| ***Driving Time*** *(per 15-minute increase)* |  | **-0.06 (-0.08, -0.04)** | **-0.06 (-0.08, -0.04)** | **-0.08 (-0.13, -0.02)** |  |  |  |  |  |  |
| **Interaction, Race * Driving Travel Time** |  |  |  |  |  |  |  |  |  |  |
| **NHW** |  |  |  | REFERENCE |  |  |  |  |  |  |
| **NHB** |  |  |  | 0.01 (-0.06, 0.08) |  |  |  |  |  |  |
| **Hispanic** |  |  |  | -0.07 (-0.15, 0.00) |  |  |  |  |  |  |
| **AANHPI*** |  |  |  | -0.03 (-0.12, 0.06) |  |  |  |  |  |  |
| **Chinese** |  |  |  | 0.04 (-0.15, 0.23) |  |  |  |  |  |  |
| **Japanese** |  |  |  | -0.01 (-0.28, 0.25) |  |  |  |  |  |  |
| **Filipino** |  |  |  | -0.03 (-0.18, 0.12) |  |  |  |  |  |  |
| **Korean** |  |  |  | 0.05 (-0.11, 0.20) |  |  |  |  |  |  |
| **Vietnamese** |  |  |  | -0.27 (-0.57, 0.03) |  |  |  |  |  |  |
| **Other Asian** |  |  |  | 0.04 (-0.11, 0.20) |  |  |  |  |  |  |
| ***Public Transit Time*** *(per 15-minutes increase)* |  |  |  |  |  | **-0.02 (-0.03, -0.01)** | **-0.02 (-0.03, -0.01)** | -0.02 (-0.05, 0.00) |  |  |
| **Interaction, Race * Public Transit Travel Time** |  |  |  |  |  |  |  |  |  |  |
| **NHW** |  |  |  |  |  |  |  | REFERENCE |  |  |
| **NHB** |  |  |  |  |  |  |  | 0.03 (-0.01, 0.06) |  |  |
| **Hispanic** |  |  |  |  |  |  |  | -0.01 (-0.05, 0.03) |  |  |
| **AANHPI*** |  |  |  |  |  |  |  | -0.02 (-0.05, 0.02) |  |  |
| **Chinese** |  |  |  |  |  |  |  | -0.03 (-0.12, 0.06) |  |  |
| **Japanese** |  |  |  |  |  |  |  | 0.03 (-0.09, 0.15) |  |  |
| **Filipino** |  |  |  |  |  |  |  | -0.04 (-0.11, 0.03) |  |  |
| **Korean** |  |  |  |  |  |  |  | -0.04 (-0.14, 0.05) |  |  |
| **Vietnamese** |  |  |  |  |  |  |  | -0.04 (-0.12, 0.04) |  |  |
| **Other Asian** |  |  |  |  |  |  |  | 0.06 (-0.03, 0.16) |  |  |
| **Outcome: Delayed GCT Driving Travel Time n = 18,325; Public Transit Travel Time n = 9,156** | | | | | | | | | |  |
| ***Race*** |  |  |  |  |  |  |  |  |  |  |
| **NHW** | REFERENCE |  | REFERENCE | REFERENCE | REFERENCE |  | REFERENCE | REFERENCE | REFERENCE |  |
| **NHB** | **0.15 (0.09, 0.21)** |  | **0.15 (0.08, 0.21)** | **0.15 (0.09, 0.21)** | **0.16 (0.07, 0.25)** |  | **0.13 (0.06, 0.21)** | **0.14 (0.07, 0.22)** | **0.17 (0.09, 0.25)** |  |
| **Hispanic** | **0.08 (0.03, 0.14)** |  | **0.08 (0.03, 0.13)** | **0.08 (0.02, 0.13)** | 0.06 (-0.01, 0.13) |  | **0.10 (0.03, 0.18)** | **0.10 (0.03, 0.18)** | **0.11 (0.03, 0.19)** |  |
| **AANHPI*** | 0.02 (-0.03, 0.08) |  | 0.02 (-0.04, 0.07) | 0.02 (-0.04, 0.07) | 0.00 (-0.07, 0.08) |  | **0.07 (0.00, 0.14)** | 0.07 (-0.01, 0.14) | 0.06 (-0.02, 0.14) |  |
| **Chinese** | -0.04 (-0.14, 0.06) |  | -0.04 (-0.14, 0.05) | -0.06 (-0.17, 0.04) | -0.13 (-0.29, 0.03) |  | 0.01 (-0.10, 0.13) | 0.01 (-0.12, 0.15) | 0.01 (-0.16, 0.19) |  |
| **Japanese** | 0.03 (-0.16, 0.21) |  | 0.02 (-0.16, 0.20) | 0.05 (-0.14, 0.24) | 0.13 (-0.16, 0.43) |  | 0.10 (-0.14, 0.34) | 0.12 (-0.12, 0.36) | 0.16 (-0.10, 0.43) |  |
| **Filipino** | **0.18 (0.10, 0.27)** |  | **0.18 (0.09, 0.27)** | **0.19 (0.10, 0.28)** | **0.24 (0.14, 0.35)** |  | **0.19 (0.08, 0.31)** | **0.20 (0.08, 0.31)** | **0.24 (0.12, 0.35)** |  |
| **Korean** | -0.01 (-0.21, 0.18) |  | -0.01 (-0.21, 0.19) | -0.03 (-0.24, 0.18) | -0.15 (-0.48, 0.18) |  | -0.02 (-0.25, 0.22) | -0.02 (-0.25, 0.22) | -0.06 (-0.32, 0.21) |  |
| **Vietnamese** | 0.11 (-0.03, 0.25) |  | 0.10 (-0.03, 0.24) | 0.06 (-0.09, 0.22) | -0.04 (-0.30, 0.21) |  | **0.22 (0.06, 0.38)** | 0.13 (-0.05, 0.31) | 0.02 (-0.21, 0.25) |  |
| **Other Asian** | **-0.17 (-0.34, 0.00)** |  | -0.18 (-0.35, -0.01) | -0.17 (-0.34, 0.01) | -0.13 (-0.36, 0.09) |  | -0.07 (-0.28, 0.14) | -0.10 (-0.34, 0.14) | -0.14 (-0.44, 0.17) |  |
| ***Driving Time*** *(per 15-minute increase)* |  | **-0.03 (-0.04, -0.02)** | **-0.03 (-0.04, -0.02)** | **-0.04 (-0.08, -0.01)** |  |  |  |  |  |  |
| **Interaction, Race * Driving Travel Time** |  |  |  |  |  |  |  |  |  |  |
| **NHW** |  |  |  | REFERENCE |  |  |  |  |  |  |
| **NHB** |  |  |  | 0.01 (-0.04, 0.06) |  |  |  |  |  |  |
| **Hispanic** |  |  |  | -0.02 (-0.06, 0.02) |  |  |  |  |  |  |
| **AANHPI*** |  |  |  | -0.01 (-0.06, 0.03) |  |  |  |  |  |  |
| **Chinese** |  |  |  | -0.07 (-0.15, 0.02) |  |  |  |  |  |  |
| **Japanese** |  |  |  | 0.08 (-0.09, 0.26) |  |  |  |  |  |  |
| **Filipino** |  |  |  | 0.05 (0.00, 0.10) |  |  |  |  |  |  |
| **Korean** |  |  |  | -0.12 (-0.31, 0.07) |  |  |  |  |  |  |
| **Vietnamese** |  |  |  | -0.11 (-0.26, 0.04) |  |  |  |  |  |  |
| **Other Asian** |  |  |  | 0.03 (-0.08, 0.15) |  |  |  |  |  |  |
| ***Public Transit Time*** *(per 15-minutes increase)* |  |  |  |  |  | **-0.01 (-0.01, 0.00)** | **-0.01 (-0.01, 0.00)** | -0.02 (-0.04, 0.00) |  |  |
| **Interaction, Race * Public Transit Travel Time** |  |  |  |  |  |  |  |  |  |  |
| **NHW** |  |  |  |  |  |  |  | REFERENCE |  |  |
| **NHB** |  |  |  |  |  |  |  | **0.02 (0.01, 0.04)** |  |  |
| **Hispanic** |  |  |  |  |  |  |  | **0.01 (-0.01, 0.03)** |  |  |
| **AANHPI*** |  |  |  |  |  |  |  | -0.01 (-0.03, 0.02) |  |  |
| **Chinese** |  |  |  |  |  |  |  | 0.00 (-0.06, 0.06) |  |  |
| **Japanese** |  |  |  |  |  |  |  | 0.04 (-0.04, 0.13) |  |  |
| **Filipino** |  |  |  |  |  |  |  | **0.04 (0.01, 0.06)** |  |  |
| **Korean** |  |  |  |  |  |  |  | -0.04 (-0.11, 0.03) |  |  |
| **Vietnamese** |  |  |  |  |  |  |  | -0.11 (-0.18, -0.04) |  |  |
| **Other Asian** |  |  |  |  |  |  |  | -0.04 (-0.15, 0.07) |  |  |
| *****Separate model with aggregate AANHPI which include NHPI and Asian Indians. | | | | | | | | | | |
| **^a^**Adjusted for age, year of diagnosis, stage at diagnosis, sex, insurance, marital status, cancer approved program, and rural-urban continuum code | | | | | | | | | | |
| **^b^**Estimates for race represent race effects as modified by a 15-minute increase in travel time (with product term to capture effect modification by travel time). | | | | | | | | | | |
