# Supplementary material for: The impact of patient travel time on disparities in treatment for early stage lung cancer in California
Source: PLoS One. 2022 Oct 5;17(10):e0272076. doi: 10.1371/journal.pone.0272076 (PMC9534452; doi:10.1371/journal.pone.0272076)
Supplement: S2 Table — (DOCX) [file pone.0272076.s002.docx]

**S2 Table.** **Effect of travel time to treatment facilities on socioeconomic disparities in undertreatment and delayed GCT.** Betas and 95% CIs for Model 15 through Model 28 were adjusted for age, year of diagnosis, stage at diagnosis, sex, race/ethnicity, insurance, marital status, cancer approved program, and rural-urban continuum code. Model 15 regressed undertreatment on nSES. Model 16 regressed undertreatment on driving time. Model 17 combined Model 15 and Model 16. Model 18 extended Model 17 by adding an interaction term between nSES and driving time. Model 19 regressed undertreatment on public transit time. Model 20 combined Model 15 and Model 19. Model 21 extended Model 20 by adding an interaction term between nSES and public transit time. Model 22 regressed delayed GCT on nSES. Model 23 regressed delayed GCT on driving time. Model 24 combined Model 22 and Model 23. Model 25 extended Model 24 by adding an interaction term between nSES and driving time. Model 26 regressed delayed GCT on public transit time. Model 27 combined Model 22 and Model 26. Model 28 extended Model 27 by adding an interaction term between nSES and public transit time. Estimates for nSES represent nSES effects as modified by a 15-minute increase in travel time (with product term to capture effect modification by travel time).

| **Variable** | **Model 15^a^** | **Model 16^a^** | **Model 17^a^** | **Model 18^a^** | **Model 18 Summary^b^** | **Model 19^a^** | **Model 20^a^** | **Model 21^a^** | **Model 21 Summary^b^** |
| --- | --- | --- | --- | --- | --- | --- | --- | --- | --- |
| **Outcome: Undertreatment Driving Travel Time n = 22,821; Public Transit Travel Time n = 11,607** | | | | | | | | | |
| ***Neighborhood SES*** |  |  |  |  |  |  |  |  |  |
| **Highest** | REFERENCE |  | REFERENCE | REFERENCE | REFERENCE |  | REFERENCE | REFERENCE | REFERENCE |
| **Upper-Middle** | 0.03 (-0.05, 0.12) |  | 0.04 (-0.04, 0.12) | 0.04 (-0.04, 0.13) | 0.06 (-0.07, 0.19) |  | 0.02 (-0.10, 0.13) | 0.03 (-0.09, 0.15) | 0.05 (-0.09, 0.19) |
| **Middle** | **0.13 (0.05, 0.21)** |  | **0.14 (0.06, 0.22)** | **0.13 (0.05, 0.22)** | 0.12 (-0.01, 0.25) |  | **0.13 (0.01, 0.24)** | **0.13 (0.01, 0.25)** | **0.14 (0.01, 0.27)** |
| **Lower-Middle** | **0.23 (0.15, 0.31)** |  | **0.24 (0.15, 0.32)** | **0.24 (0.15, 0.32)** | **0.24 (0.12, 0.36)** |  | **0.26 (0.15, 0.37)** | **0.26 (0.15, 0.38)** | **0.27 (0.15, 0.40)** |
| **Lowest** | **0.30 (0.22, 0.39)** |  | **0.31 (0.22, 0.39)** | **0.30 (0.21, 0.39)** | **0.28 (0.15, 0.42)** |  | **0.33 (0.21, 0.45)** | **0.33 (0.21, 0.45)** | **0.33 (0.20, 0.47)** |
| ***Driving Time*** *(per 15-minute increase)* |  | **-0.06 (-0.08, -0.04)** | **-0.06 (-0.08, -0.04)** | **-0.06 (-0.08, -0.04)** |  |  |  |  |  |
| **Interaction, nSES * Driving Travel Time** |  |  |  |  |  |  |  |  |  |
| **Highest** |  |  |  | REFERENCE |  |  |  |  |  |
| **Upper-Middle** |  |  |  | 0.02 (-0.06, 0.10) |  |  |  |  |  |
| **Middle** |  |  |  | -0.02 (-0.09, 0.06) |  |  |  |  |  |
| **Lower-Middle** |  |  |  | 0.00 (-0.07, 0.07) |  |  |  |  |  |
| **Lowest** |  |  |  | -0.02 (-0.09, 0.06) |  |  |  |  |  |
| ***Public Transit Time*** *(per 15-minutes increase)* |  |  |  |  |  | **-0.02 (-0.03, -0.01)** | **-0.02 (-0.03, -0.01)** | **-0.02 (-0.03, -0.01)** |  |
| **Interaction, nSES * Public Transit Travel Time** |  |  |  |  |  |  |  |  |  |
| **Highest** |  |  |  |  |  |  |  | REFERENCE |  |
| **Upper-Middle** |  |  |  |  |  |  |  | 0.02 (-0.02, 0.07) |  |
| **Middle** |  |  |  |  |  |  |  | 0.01 (-0.03, 0.04) |  |
| **Lower-Middle** |  |  |  |  |  |  |  | 0.01 (-0.03, 0.05) |  |
| **Lowest** |  |  |  |  |  |  |  | 0.00 (-0.04, 0.04) |  |
| **Outcome: Delayed GCT Driving Travel Time n = 18,471; Public Transit Travel Time n = 9,227** | | | | | | | | | |
| ***Neighborhood SES*** |  |  |  |  |  |  |  |  |  |
| **Highest** | REFERENCE |  | REFERENCE | REFERENCE | REFERENCE |  | REFERENCE | REFERENCE | REFERENCE |
| **Upper-Middle** | **0.19 (0.14, 0.25)** |  | **0.19 (0.14, 0.25)** | **0.20 (0.14, 0.26)** | **0.23 (0.15, 0.30)** |  | **0.12 (0.05, 0.20)** | **0.12 (0.05, 0.20)** | **0.12 (0.04, 0.21)** |
| **Middle** | **0.24 (0.18, 0.29)** |  | **0.24 (0.18, 0.29)** | **0.24 (0.19, 0.30)** | **0.26 (0.18, 0.33)** |  | **0.20 (0.13, 0.28)** | **0.20 (0.12, 0.28)** | **0.19 (0.11, 0.28)** |
| **Lower-Middle** | **0.27 (0.22, 0.33)** |  | **0.27 (0.22, 0.33)** | **0.28 (0.23, 0.34)** | **0.32 (0.25, 0.40)** |  | **0.19 (0.11, 0.26)** | **0.19 (0.11, 0.27)** | **0.19 (0.11, 0.27)** |
| **Lowest** | **0.32 (0.26, 0.38)** |  | **0.32 (0.26, 0.38)** | **0.33 (0.27, 0.39)** | **0.37 (0.29, 0.45)** |  | **0.24 (0.15, 0.32)** | **0.24 (0.15, 0.32)** | **0.24 (0.15, 0.33)** |
| ***Driving Time*** *(per 15-minute increase)* |  | **-0.03 (-0.04, -0.02)** | **-0.03 (-0.04, -0.02)** | **-0.03 (-0.05, -0.02)** |  |  |  |  |  |
| **Interaction, nSES * Driving Travel Time** |  |  |  |  |  |  |  |  |  |
| **Highest** |  |  |  | REFERENCE |  |  |  |  |  |
| **Upper-Middle** |  |  |  | 0.03 (-0.01, 0.07) |  |  |  |  |  |
| **Middle** |  |  |  | 0.01 (-0.03, 0.05) |  |  |  |  |  |
| **Lower-Middle** |  |  |  | **0.04 (0.00, 0.08)** |  |  |  |  |  |
| **Lowest** |  |  |  | 0.04 (-0.01, 0.08) |  |  |  |  |  |
| ***Public Transit Time*** *(per 15-minutes increase)* |  |  |  |  |  | **-0.01 (-0.01, 0.00)** | **-0.01 (-0.01, 0.00)** | -0.01 (-0.01, 0.00) |  |
| **Interaction, nSES * Public Transit Travel Time** |  |  |  |  |  |  |  |  |  |
| **Highest** |  |  |  |  |  |  |  | REFERENCE |  |
| **Upper-Middle** |  |  |  |  |  |  |  | 0.00 (-0.03, 0.03) |  |
| **Middle** |  |  |  |  |  |  |  | -0.01 (-0.04, 0.01) |  |
| **Lower-Middle** |  |  |  |  |  |  |  | 0.00 (-0.02, 0.03) |  |
| **Lowest** |  |  |  |  |  |  |  | 0.00 (-0.03, 0.02) |  |
| *****Separate model with aggregate AANHPI which include NHPI and Asian Indians. | | | | | | | | | |
| **^a^**Adjusted for age, year of diagnosis, stage at diagnosis, sex, race/ethnicity, insurance, marital status, cancer approved program, and rural-urban continuum code | | | | | | | | | |
| **^b^**Estimates for nSES represent nSES effects as modified by a 15-minute increase in travel time (with product term to capture effect modification by travel time). | | | | | | | | | |
