# Supplementary material for: The impact of patient travel time on disparities in treatment for early stage lung cancer in California
Source: PLoS One. 2022 Oct 5;17(10):e0272076. doi: 10.1371/journal.pone.0272076 (PMC9534452; doi:10.1371/journal.pone.0272076)
Supplement: S3 Table — (DOCX) [file pone.0272076.s003.docx]

**S3 Table.** **Driving and public transit times, stratified by neighborhood socioeconomic status.**

|  | **All** | **Patient Neighborhood Socioeconomic Status** | | | | |
| --- | --- | --- | --- | --- | --- | --- |
|  |  | **Lowest (n = 3243)** | **Lower-middle (n = 4494)** | **Middle (n = 4927)** | **Upper-Middle (n = 5025)** | **Highest (n = 5132)** |
| **Exposure Variables** | **n (%) or *Mean (SD)** | | | | | |
| ***Driving Travel Times**** | 26.0 (26.5) | 26.3 (27.5) | 27.3 (29.3) | 27.2 (28.9) | 25.8 (26) | 23.9 (20.3) |
| < 15 minutes | 8703 (38.1) | 1363 (42.0) | 1804 (40.1) | 1851 (37.6) | 1855 (36.9) | 1830 (35.7) |
| 15 - 30 minutes | 8345 (36.6) | 1066 (32.9) | 1515 (33.7) | 1729 (35.1) | 1896 (37.7) | 2139 (41.7) |
| 30- 60 minutes | 4033 (17.7) | 503 (15.5) | 759 (16.9) | 938 (19.0) | 938 (18.7) | 895 (17.4) |
| ≥ 60 minutes | 1740 (7.6) | 311 (9.6) | 416 (9.3) | 409 (8.3) | 336 (6.7) | 268 (5.2) |
| ***Public Transit Travel***  ***Times****** | 68.6 (66.2) | 68.5 (78.0) | 69.5 (71.8) | 68.7 (66.5) | 67.6 (63.5) | 68.6 (49.7) |
| < 15 minutes | 476 (2.1) | 80 (2.5) | 96 (2.1) | 93 (1.9) | 122 (2.4) | 85 (1.7) |
| 15 - 30 minutes | 1891 (8.3) | 328 (10.1) | 408 (9.1) | 413 (8.4) | 417 (8.3) | 325 (6.3) |
| 30- 60 minutes | 4186 (18.3) | 733 (22.6) | 912 (20.3) | 880 (17.9) | 870 (17.3) | 791 (15.4) |
| ≥ 60 minutes | 5054 (22.2) | 749 (23.1) | 1046 (23.3) | 1076 (21.8) | 1093 (21.8) | 1090 (21.2) |
| Unavailable | 11214 (49.1) | 1353 (41.7) | 2032 (45.2) | 2465 (50.0) | 2523 (50.2) | 2841 (55.4) |
