# Supplementary material for: The impact of patient travel time on disparities in treatment for early stage lung cancer in California
Source: PLoS One. 2022 Oct 5;17(10):e0272076. doi: 10.1371/journal.pone.0272076 (PMC9534452; doi:10.1371/journal.pone.0272076)
Supplement: S4 Table — (DOCX) [file pone.0272076.s004.docx]

**S4 Table.** **Sensitivity Analysis: Risk Ratios (RR) and 95% Confidence Intervals (CI) for race/ethnicity and neighborhood socioeconomic status (nSES) representing that effect as modified by a 15-minute increase in driving time calculate using the *gmapsdistance* function.**

|  | **Outcome: Undertreatment** | | **Outcome: Delayed GCT** | |
| --- | --- | --- | --- | --- |
|  | **Exposure: Driving Time** | | | |
|  | **Model 4 Summary^a^** | **Model 18 Summary^b^** | **Model 11 Summary^a^** | **Model 21 Summary^b^** |
| **Effect Modifier** | **RR (95 CI)** |  | **RR (95 CI)** |  |
| ***Race/Ethnicity*** |  |  |  |  |
| **non-Hispanic White** | REFERENCE |  | REFERENCE |  |
| **non-Hispanic Black** | **1.21 (1.03, 1.42)** |  | **1.17 (1.05, 1.29)** |  |
| **Hispanic** | 1.08 (0.94, 1.26) |  | 1.06 (0.98, 1.14) |  |
| **AANHPI*** | 1.02 (0.84, 1.24) |  | 0.99 (0.90, 1.09) |  |
| **Chinese** | 0.95 (0.66, 1.37) |  | 0.78 (0.62, 0.97) |  |
| **Japanese** | 0.87 (0.53, 1.45) |  | 1.19 (0.84, 1.69) |  |
| **Filipino** | 1.07 (0.78, 1.47) |  | **1.30 (1.16, 1.46)** |  |
| **Korean** | 1.25 (0.84, 1.84) |  | 0.77 (0.45, 1.31) |  |
| **Vietnamese** | 0.87 (0.45, 1.68) |  | 1.00 (0.73, 1.37) |  |
| **Other Asian** | 1.31 (0.95, 1.79) |  | 0.82 (0.61, 1.10) |  |
| ***Neighborhood SES*** |  |  |  |  |
| **Highest** |  | REFERENCE |  | REFERENCE |
| **Upper-Middle** |  | 1.09 (0.93, 1.27) |  | **1.28 (1.17, 1.40)** |
| **Middle** |  | 1.14 (0.98, 1.33) |  | **1.32 (1.21, 1.44)** |
| **Lower-Middle** |  | **1.28 (1.10, 1.48)** |  | **1.41 (1.29, 1.54)** |
| **Lowest** |  | **1.35 (1.15, 1.59)** |  | **1.48 (1.35, 1.62)** |
| *****Separate model with aggregate AANHPI which include NHPI and Asian Indians. | | | | |
| ^a^ Risk Ratio (Exponentiated Estimate) for Race/Ethnicity represents Race/Ethnicity effect as modified by a 15-minute increase in travel time (with product term to capture effect modification by travel time, adjusted for age, year of diagnosis, stage at diagnosis, sex, insurance, marital status, cancer approved program, and rural-urban continuum code. | | | | |
| ^b^ Risk Ratio (Exponentiated Estimates) for nSES represent nSES effects as modified by a 15-minute increase in travel time (with product term to capture effect modification by travel time), adjusted for age, year of diagnosis, stage at diagnosis, sex, race/ethnicity, insurance, marital status, cancer approved program, and rural-urban continuum code | | | | |
|  |  |  |  |  |
